# Healthcare utilization, medical expenditure, and mortality in Korean patients with pulmonary hypertension

**DOI:** 10.1186/s12890-019-0945-0

**Published:** 2019-10-30

**Authors:** In-Chang Hwang, Goo-Yeong Cho, Hong-Mi Choi, Yeonyee E. Yoon, Jin Joo Park, Jun-Bean Park, Seung-Pyo Lee, Hyung-Kwan Kim, Yong-Jin Kim, Dae-Won Sohn

**Affiliations:** 1 Department of Cardiology, Cardiovascular Center, Seoul National University Bundang Hospital, Seongnam, Gyeonggi South Korea; 2 Department of Internal Medicine, Seoul National University College of Medicine, Seoul, South Korea; 3 Division of Cardiology, Hallym Sacred Heart Hospital, Hallym University College of Medicine, Anyang, South Korea; 4 Cardiovascular Center and Department of Internal Medicine, Seoul National University Hospital, Seoul, South Korea

**Keywords:** Pulmonary hypertension, Healthcare utilization, Mortality

## Abstract

**Background:**

Limited data exists regarding healthcare utilization, medical expenses, and prognosis of pulmonary hypertension (PH) according to the World Health Organization (WHO) classification. We aimed to investigate mortality risk, healthcare utilization and medical expenditure in patients with PH across the five diagnostic subgroups.

**Methods:**

We identified 2185 patients with PH, defined as peak tricuspid regurgitation velocity > 3.4 m/sec, among the consecutive patients referred for echocardiography between 2009 and 2015. Using diagnostic codes, medical records, and echocardiographic findings, the enrolled patients were classified according to the five subgroups by WHO classification. Healthcare utilization, costs, and all-cause mortality were assessed.

**Results:**

Diagnostic subgroups of PH demonstrated significantly different clinical features. During a median of 32.4 months (interquartile range, 16.2–57.8), 749 patients (34.3%) died. Mortality risk was the lowest in group II (left heart disease) and highest in group III (chronic lung disease). The etiologies of pulmonary arterial hypertension (PAH) had significant influence on the mortality risk in group I, showing the worst prognosis in PAH associated with connective tissue disease. Medical expenditure and healthcare utilization were different between the PH subgroups: groups II and V had more hospitalizations and medical expenses than other groups. Regardless of PH subgroups, the severity of PH was associated with higher mortality risk, more healthcare utilization and medical expenditure.

**Conclusions:**

Significant differences in clinical features and prognostic profiles between PH subgroups reflect the differences in pathophysiology and clinical consequences. Our findings highlight the importance of comprehensive understanding of PH according to the etiology and its severity.

## Background

Pulmonary hypertension (PH) is a frequently encountered clinical condition. Several cohort studies reported that the prevalence of PH is higher than previously recognized: ranges from 2.6% among general population, [1] to 9.1% among those who were referred for echocardiography [2]. In addition, the presence of PH is associated with higher risk of cardiovascular events.

PH is categorized into 5 diagnostic subgroups of different etiology: pulmonary arterial hypertension (PAH; group I), PH due to left-sided heart disease (PH-LHD; group II), PH due to lung disease or hypoxemia (PH-Lung; group III), chronic thromboembolic PH (CTEPH; group IV), and PH of miscellaneous causes (PH-Miscellaneous) [3]. Until now, there have been several studies that compared the overall incidence of PH according to the WHO classification [2, 4, 5]. But most of these studies limited their scope to idiopathic or associated PAH, but not other subgroups [6]. Moreover, there has been no study that compared healthcare utilization and medical expenditure according to the WHO classification of PH.

In this study, we aimed to describe the clinical characteristics of the five diagnostic subgroups of PH according to the WHO classification, to compare the risk of mortality of PH patients between the subgroups, and to investigate whether the healthcare utilization and medical costs are different between the five subgroups.

## Methods

### Study population and WHO classification

The study was carried out according to the principles of the Declaration of Helsinki and approved by the Clinical Research Institute of Seoul National University Bundang Hospital.

We retrospectively identified a total of 272,688 echocardiography exams performed at Seoul National University Bundang Hospital and Seoul National University Hospital between 2009 and 2015. We used a peak tricuspid regurgitant (TR) flow velocity > 3.4 m/sec for echocardiographic diagnosis of PH, [3] and identified 2843 patients with PH. Then, study population was classified according to the five diagnostic subgroups of PH, using a 2-step process: at first, we categorized the total study population using the comorbidities that were identified by diagnostic codes according to the International Statistical Classification of Diseases and Related Health Problems, 10th revision (ICD-10) (Additional file 1), and by reviewing detailed descriptions in the electronic medical record. Echocardiographic findings of grade II or III diastolic dysfunction or septal E/e’ ratio > 15 were also used for the categorization of group II PH (PH-LHD) [7]. When a patient has diagnostic codes, echocardiographic findings, or other characteristics satisfying more than 1 subgroup of PH, the patient’s electronic medical record was reviewed for differentiation. Two reviewers independently assessed the study population according to WHO classification, and cases of disagreement within the reviewers were resolved through discussion.

Patients with uncertain classification and those with prior diagnosis of malignancy were excluded (*n* = 653), in order to avoid any impacts from malignancy on the medical expenditure for diagnosis and management, as well as on the prognosis. Finally, a total of 2185 patients were included in the analysis.

### Echocardiography

All echocardiography images were obtained in accordance with current guidelines [8]. LV end-systolic and end-diastolic volumes and LV ejection fraction (LVEF) were calculated using the biplane Simpson method. Left atrial (LA) volume was calculated by the biplane area-length method [8]. Pulmonary artery systolic pressure (PASP) was estimated by summing the peak systolic trans-tricuspid pressure gradient calculated from peak velocity of TR and right atrial pressure (RAP) estimated by diameter and inspiratory collapsibility of inferior vena cava (IVC): the RAP was estimated as 3 mmHg in patients with IVC diameter < 2.1 cm that collapses > 50% with sniffing, 8 mmHg in those with IVC diameter > 2.1 cm that collapses > 50% and in those with IVC diameter < 2.1 cm that collapses < 50%, and 15 mmHg in those with IVC diameter > 2.1 cm that collapses < 50% [3]. Severity of PH was estimated using the median PASP value of total study population (62 mmHg) or arbitrary cutoff values of PASP (60 and 70 mmHg).

### Study outcomes

All-cause mortality during follow-up was assessed until January 2017. For the confirmation of death, we used the database from the Korean Ministry of Security and Public Administration. Healthcare utilization and medical costs of each patient were assessed from July 2009 to December 2016, and were compared between the subgroups of PH. The number of outpatient department (OPD) visits, number of hospitalizations, and total duration of hospitalizations were assessed. Expenditure for medication, diagnostic testing, and total costs including medication, diagnostic testing, therapeutic measures, and hospital stay were compiled using reimbursement database of the National Health Insurance program. Medical costs were assessed in Korean Won (KRW), and were translated into US dollars ($USD) using a currency exchange rate of 1123 KRW/$USD. In order to avoid the potential influence from the hospitalizations and related medical costs that have been intensively occurred early after enrollment, patients with follow-up duration < 6 months were excluded from the analysis for healthcare utilization and medical costs.

### Statistical analysis

Continuous variables are presented as mean ± standard deviation (SD) or median with interquartile ranges (IQR; Q1–Q3); categorical variables are expressed as percentages. Differences in continuous variables were assessed by Student’s t-test, and categorical variables by Chi-square test or Fisher’s exact test. Cox proportional hazard regression model with backward selection method were used to compare times to the death. Adjusted hazard ratios (HRs) for all-cause mortality were calculated across the PH subgroups. Univariable markers with *P* values < 0.100 entered multivariable analysis. All statistical analyses were performed using SPSS 20.0 (IBM Corp., NY, USA).

## Results

### Baseline characteristics

Table 1 shows baseline characteristics of the study population according to the WHO classification of PH. The mean age of total study population was 67.7 ± 14.9 years, and 51.4% were female. Patients of group I were younger than other groups, and patients of group III were older than other groups. More than two-thirds of patients were female in group I and group IV, whereas more than two-thirds were male in group III. Prevalence of comorbidities showed significant differences between the WHO groups of PH: group II had a higher prevalence of hypertension and dyslipidemia, and most of the group V had chronic kidney disease (CKD) or end-stage renal disease (ESRD).

**Table 1:** Baseline characteristics

	Total study population	Group I (PAH)	Group II (PH-LHD)	Group III (PH-Lung)	Group IV (CTEPH)	Group V (PH-Miscellaneous)
	(*n* = 2185)	(*n* = 333)	(*n* = 1189)	(*n* = 335)	(*n* = 102)	(*n* = 226)
Age (years)	67.7 ± 14.9	56.5 ± 17.6	70.0 ± 13.6	72.5 ± 10.6	68.4 ± 14.8	64.4 ± 14.4
Female sex	1123 (51.4%)	227 (68.2%)	611 (51.4%)	110 (32.8%)	71 (69.6%)	104 (46.0%)
BMI (kg/m^2^)	22.9 ± 3.9	22.6 ± 4.5	23.1 ± 3.7	21.5 ± 3.9	24.9 ± 3.8	22.5 ± 3.5
Comorbidities						
Hypertension	638 (29.2%)	83 (24.9%)	389 (32.7%)	66 (19.7%)	27 (26.5%)	73 (32.3%)
Diabetes mellitus	611 (28.0%)	45 (13.5%)	385 (32.4%)	63 (18.8%)	17 (16.7%)	101 (44.7%)
Dyslipidemia	288 (13.2%)	26 (7.8%)	200 (16.8%)	25 (7.5%)	13 (12.7%)	24 (10.6%)
CKD	556 (25.4%)	37 (11.1%)	270 (22.7%)	27 (8.1%)	5 (4.9%)	217 (96.0%)
Chronic hepatitis	307 (14.1%)	18 (5.4%)	202 (17.0%)	58 (17.3%)	10 (9.8%)	19 (8.4%)
Laboratory findings						
Hemoglobin (g/dL)	11.8 ± 2.4	12.7 ± 2.8	11.7 ± 2.2	12.6 ± 2.4	12.1 ± 2.4	9.9 ± 1.9
Total cholesterol (mg/dL)	154.9 ± 42.8	154.0 ± 41.6	155.5 ± 42.3	150.8 ± 41.6	172.2 ± 48.9	150.8 ± 44.2
Triglyceride (mg/dL)	108.5 ± 66.6	111.7 ± 58.8	106.4 ± 61.6	99.6 ± 52.1	170.6 ± 149.3	102.0 ± 51.8
HDL-cholesterol (mg/dL)	45.0 ± 14.4	46.2 ± 15.0	44.4 ± 13.9	48.1 ± 17.2	44.9 ± 15.1	44.5 ± 13.2
LDL-cholesterol (mg/dL)	91.7 ± 33.3	95.9 ± 31.6	90.6 ± 33.0	87.3 ± 27.4	110.5 ± 38.7	91.1 ± 37.8
BUN (mg/dL)	26.8 ± 19.9	18.1 ± 11.5	27.4 ± 18.8	19.3 ± 11.8	17.9 ± 7.8	51.2 ± 26.8
Creatinine (mg/dL)	1.9 ± 2.6	1.1 ± 1.4	1.8 ± 2.1	1.0 ± 07	0.9 ± 0.7	5.9 ± 4.0
GFR (mL/min/1.73m^2^)	80.6 ± 55.7	107.6 ± 56.7	75.2 ± 51.0	105.2 ± 53.5	108.2 ± 41.3	22.4 ± 24.7
HbA1c (%)	6.5 ± 1.2	6.4 ± 1.2	6.5 ± 1.2	6.4 ± 0.9	6.5 ± 1.2	6.6 ± 1.4
Echocardiographic findings						
LVEDD (mm)	51.0 ± 10.0	45.4 ± 8.5	54.2 ± 9.7	45.3 ± 8.2	44.9 ± 8.3	53.4 ± 7.9
LVESD (mm)	35.6 ± 11.0	29.6 ± 8.5	39.0 ± 11.1	30.1 ± 8.6	30.1 ± 8.5	37.3 ± 9.4
LVEDV (mL)	111.4 ± 61.7	86.1 ± 46.3	120.2 ± 62.5	83.1 ± 47.4	75.7 ± 39.6	81.9 ± 57.8
LVESV (mL)	63.6 ± 50.4	41.5 ± 35.0	71.0 ± 52.0	42.5 ± 36.3	36.7 ± 31.6	81.9 ± 57.8
LVEF (%)	52.5 ± 15.0	60.8 ± 8.4	47.7 ± 16.3	58.3 ± 11.0	61.4 ± 8.6	52.4 ± 12.7
LVMI (g/m^2^)	121.8 ± 45.0	101.6 ± 42.2	131.6 ± 44.5	102.2 ± 39.7	98.3 ± 41.5	131.7 ± 37.8
LA dimension (mm)	46.6 ± 12.1	41.9 ± 9.4	50.2 ± 12.3	39.2 ± 10.9	39.2 ± 9.3	48.2 ± 7.8
LAVI (mL/m^2^)	71.9 ± 54.3	56.5 ± 36.8	78.1 ± 59.4	55.0 ± 36.9	51.0 ± 34.1	73.6 ± 40.3
Mitral inflow						
E velocity (m/sec)	1.04 ± 0.51	0.80 ± 0.38	1.20 ± 0.53	0.76 ± 0.38	0.70 ± 0.41	1.09 ± 0.35
A velocity (m/sec)	0.92 ± 5.40	0.74 ± 0.37	1.05 ± 7.89	0.84 ± 0.28	0.78 ± 0.24	0.85 ± 0.33
E/A ratio	1.52 ± 2.55	1.11 ± 0.60	1.85 ± 1.25	1.27 ± 5.62	0.90 ± 0.65	1.49 ± 0.87
Deceleration time (msec)	183.3 ± 92.0	188.7 ± 65.1	182.2 ± 103.6	186.0 ± 93.4	200.6 ± 73.0	169.7 ± 64.0
Mitral annular TDI						
e’ velocity (cm/sec)	5.66 ± 2.18	6.06 ± 2.33	5.46 ± 2.15	5.97 ± 2.30	5.62 ± 1.80	5.66 ± 1.90
a’ velocity (cm/sec)	7.77 ± 3.39	8.46 ± 3.11	6.58 ± 2.91	9.98 ± 3.98	9.36 ± 2.54	7.25 ± 2.73
s’ velocity (cm/sec)	6.40 ± 2.91	7.05 ± 2.18	5.83 ± 2.27	7.39 ± 3.56	7.15 ± 2.40	6.42 ± 4.67
E/e’ ratio	20.5 ± 12.8	14.7 ± 8.6	24.5 ± 13.9	14.2 ± 9.2	13.3 ± 8.7	21.1 ± 9.6
TR Vmax (m/sec)	3.7 ± 0.3	3.8 ± 0.5	3.7 ± 0.3	3.7 ± 0.4	3.8 ± 0.4	3.7 ± 0.3
PASP (mmHg)	65.5 ± 11.9	72.3 ± 18.1	62.3 ± 9.3	64.9 ± 10.8	65.6 ± 14.0	63.3 ± 7.9
Follow-up duration (months)	32.4 (16.2–57.8)	43.7 (25.3–64.0)	34.6 (18.7–60.6)	17.7 (2.2–38.7)	29.0 (10.1–52.5)	29.5 (16.0–52.1)
All-cause death	749 (34.3%)	92 (27.6%)	344 (28.9%)	192 (57.3%)	38 (37.3%)	83 (36.7%)

Data are shown as numbers (%), mean (±standard deviation), or median (interquartile ranges; Q1-Q3)

Abbreviations: *PAH* Pulmonary arterial hypertension, *PH-LHD* Pulmonary hypertension due to left heart disease, *PH-Lung* Pulmonary hypertension due to lung disease or hypoxia, *CTEPH* Chronic thromboembolic pulmonary hypertension, *PH-Miscellaneous* Pulmonary hypertension with multifactorial mechanisms, *BMI* Body-mass index, *CKD* Chronic kidney disease, *HDL* High-density lipoprotein, *LDL* Low-density lipoprotein, *BUN* Blood urea nitrogen, *GFR* Glomerular filtration rate, *HbA1c* Hemoglobin A1c, *LVEDD* Left ventricular end-diastolic dimension, *LVESD* Left ventricular end-systolic dimension, *LVEDV* Left ventricular end-diastolic volume, *LVESV* Left ventricular end-systolic volume, *LVEF* Left ventricular ejection fraction, *LVMI* Left ventricular mass index, *LA* Left atrium, *LAVI* Left atrial volume index, *TDI* Tissue Doppler imaging, *TR* Tricuspid regurgitation, *PASP* Pulmonary artery systolic pressure

Among the 333 patients with group I PH, 145 patients (43.5%) underwent cardiac catheterization for confirmative diagnosis of PAH in Seoul National University Bundang Hospital and Seoul National University Hospital during the study period. There were 177 patients (53.2%) who were on any PAH-targeted therapy among the group I PH patients: 132 patients (39.6%) were on endothelin receptor antagonists, 88 patients (26.4%) were on prostacyclin analogues, and 110 (33.0%) patients were on phosphodiesterase type 5 inhibitors.

### Echocardiographic parameters

Mean TR velocity of total study population was 3.7 ± 0.3 m/sec, and did not differ between the five subgroups (Table 1). LVEF was markedly decreased in group II (PH-LHD; 47.7 ± 16.3%), and was higher in group IV (CTEPH; 61.4 ± 8.6%) and group I (PAH; 60.8 ± 8.4%). LV cavity size and LV mass index were larger in groups II and V than other groups, suggesting a more advanced LV remodeling. Parameters of LV diastolic function, such as LA volume index and LV filling pressure assessed by E/e’ ratio, also showed worse results in these subgroups.

### Risk of mortality according to the PH subgroups

**Fig. 1:**
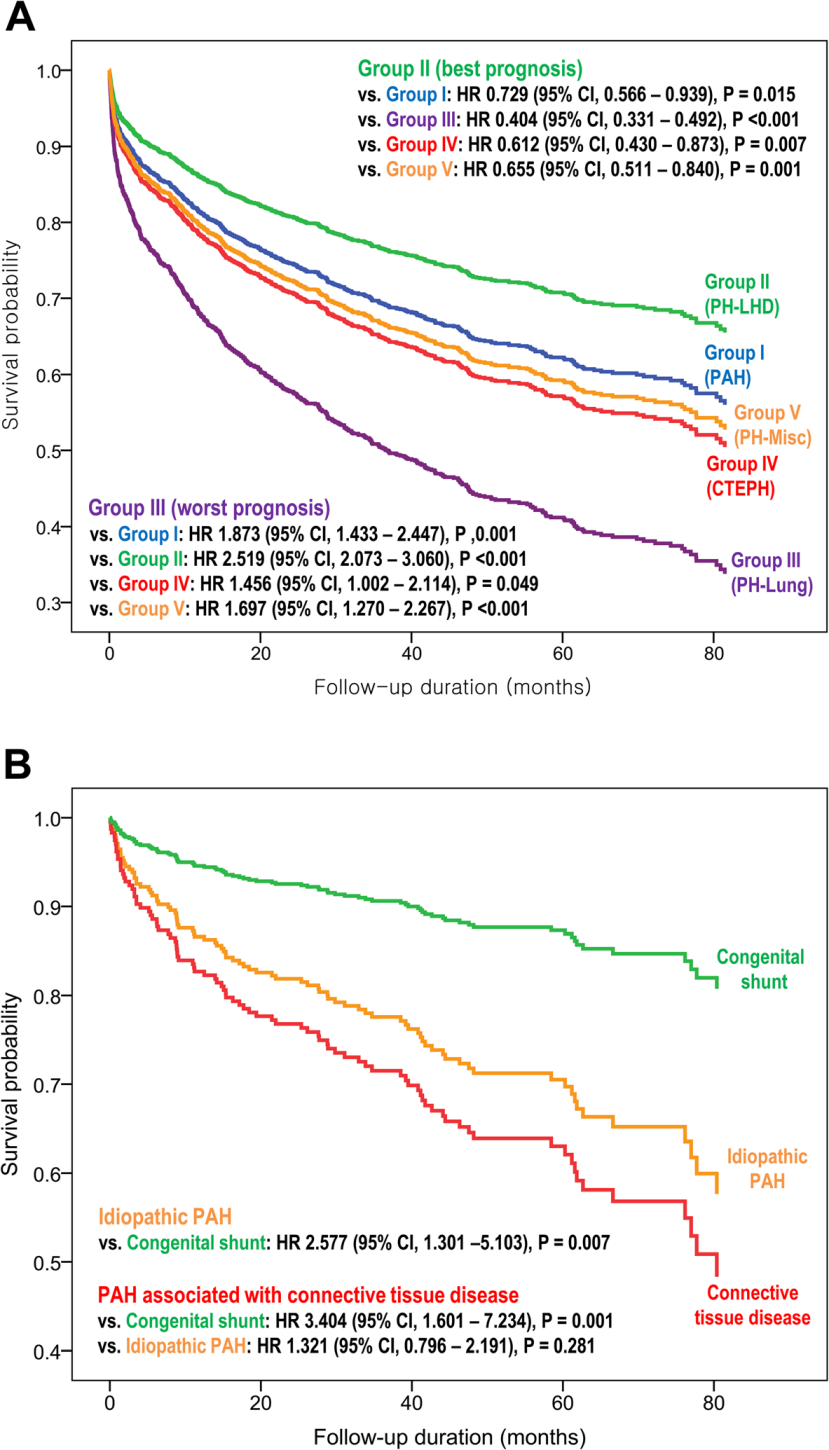
Risk-adjusted survival curves of the PH subgroups. **a** All-cause mortality-free survival by the five diagnostic subgroups of PH by WHO classification. **b** Risk-adjusted survival curves according to the etiologies of PAH. Abbreviations: PH, pulmonary hypertension; PAH, pulmonary arterial hypertension; LHD, left heart disease; CTEPH, chronic thromboembolic pulmonary hypertension; HR, hazard ratio; CI, confidence interval

During 32.4 (IQR, 16.2–57.8) months of follow-up, 749 (34.3%) deaths were observed (Table 1). We compared the all-cause mortality-free survival between the five diagnostic subgroups, adjusting the survival curves with the significant predictors for all-cause mortality (Fig. 1a). Group II had a better prognosis than other groups, while the risk of mortality was comparable between groups I, IV, and V. The mortality risk was significantly higher in group III than other groups.

The risk of all-cause mortality was also compared according to the etiology of PAH in the group I PH patients (Fig. 1b). Patients with PAH associated with connective tissue disease had the worst prognosis, followed by those with idiopathic PAH. Those with congenital shunt had relatively benign clinical course.

### Risk factors for mortality and different prognostic profiles between PH subgroups

**Fig. 2:**
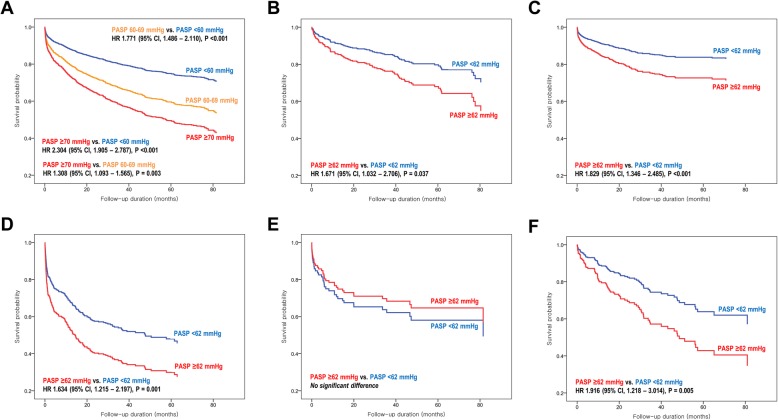
Severity of PH and all-cause mortality. Risk-adjusted all-cause mortality-free survival curves are shown, according to the severity of PH in (**a**) total study population, (**b**) group I PAH, (**c**) group II PH-LHD, (**d**) group III PH-Lung, (**e**) group IV CTEPH, and (**f**) group V PH-Miscellaneous

Given the significant differences in clinical characteristics and prognosis across the subgroups of PH, we performed Cox proportional hazard regression analysis models with backward selection method in total study population as well as in each PH subgroup. In total study population, the multivariable analyses showed that, advanced age (adjusted HR, 1.875; 95% CI, 1.390–2.529; *P* < 0.001 for age > 70 years) and male sex (adjusted HR, 1.674; 95% CI, 1.262–2.220; P < 0.001) were associated with increased risk of all-cause mortality (Table 2 and Additional file 2). A lower hemoglobin level was associated with a higher mortality risk (adjusted HR, 1.072; 95% CI, 1.010–1.138; *P* = 0.023 per − 1 g/dL of hemoglobin) (Additional file 3). The risk of mortality was proportional to the severity of PH in total study population, when stratified by PASP values of 60 and 70 mmHg (Fig. 2), or the median value of 62 mmHg (adjusted HR, 1.559; 95% CI, 1.188–2.047; *P* < 0.001) (Table 2). Of note, the elevated PASP was associated with overall increase in the risk of mortality in most subgroups (group I, II, III, and V; Fig. 2 and Table 2).

**Table 2:** Multivariate Cox proportional hazard regression analysis for each PH group

	Total study population (*n* = 2185)		Group I PH (PAH; *n* = 333)		Group II PH (PH-LHD; *n* = 1189)		Group III PH (PH-Lung; *n* = 335)		Group IV PH (CTEPH; *n* = 102)		Group V PH (PH-Miscellaneous; *n* = 226)	
	Adjusted HR (95% CI)	*P* value	Adjusted HR (95% CI)	*P* value	Adjusted HR (95% CI)	*P* value	Adjusted HR (95% CI)	*P* value	Adjusted HR (95% CI)	*P* value	Adjusted HR (95% CI)	*P* value
Age > 70 years	1.875 (1.390–2.529)	< 0.001	2.045 (1.299–3.219)	0.002	1.997 (1.335–2.988)	0.001	1.328 (0.976–1.808)	0.071			2.660 (1.702–4.158)	< 0.001
Male sex	1.674 (1.262–2.220)	< 0.001			1.765 (1.220–2.552)	0.003					1.571 (1.000–2.467)	0.050
BMI > 22.5 kg/m^2^	0.826 (0.630–1.082)	0.165			0.727 (0.535–0.986)	0.041						
Hypertension	0.810 (0.592–1.110)	0.190					0.645 (0.439–0.948)	0.026				
DM	0.864 (0.631–1.182)	0.360										
Dyslipidemia	0.970 (0.643–1.463)	0.884										
Hemoglobin (per − 1 g/dL)	1.072 (1.010–1.138)	0.023	1.100 (1.011–1.195)	0.026	1.135 (1.054–1.224)	0.001	1.107 (1.040–1.181)	0.002	1.145 (1.003–1.307)	0.045		
GFR < 60 mL/min/1.73m^2^	1.291 (0.936–1.790)	0.120	1.689 (0.945–3.021)	0.077								
LVEDV > 100 mL	1.213 (0.890–1.654)	0.221			1.514 (1.057–2.168)	0.024						
LVEF < 30%	1.058 ().693–1.616)	0.794			1.514 (1.069–2.226)	0.021						
LA dimension > 40 mm	0.848 (0.618–1.162)	0.305										
PASP > 62 mmHg	1.559 (1.188–2.047)	0.001	1.671 (1.032–2.706)	0.037	1.829 (1.346–2.485)	< 0.001	1.634 (1.215–2.197)	0.001			1.916 (1.218–3.014)	0.005

Multivariable Cox proportional hazard regression model with backward selection method were used. Adjusted hazard ratios (HRs) for all-cause mortality were calculated using the univariable markers with *P* values < 0.100. Variables associated with all-cause mortality in total study population (second column) were assessed in each PH subgroup

Abbreviations: same as Table 1

Risk factors for mortality showed differences between the PH subgroups (Table 2). In group I PH, advanced age, lower hemoglobin level, and elevated PASP were associated with higher mortality risk, and renal dysfunction showed a modest association with the mortality risk. In group II PH, advanced age, male sex, lower hemoglobin level, dilated LV cavity, and impaired LV systolic function appeared to be significant risk factors, while higher BMI was associated with low mortality risk. In group III PH, a lower level of hemoglobin was associated with higher mortality, whereas the presence of HTN was associated with lower mortality risk. Significant variables associated with mortality were lower hemoglobin level in group IV, and were advanced age and male sex in group V.

### Medical expenditure and healthcare utilization

**Fig. 3:**
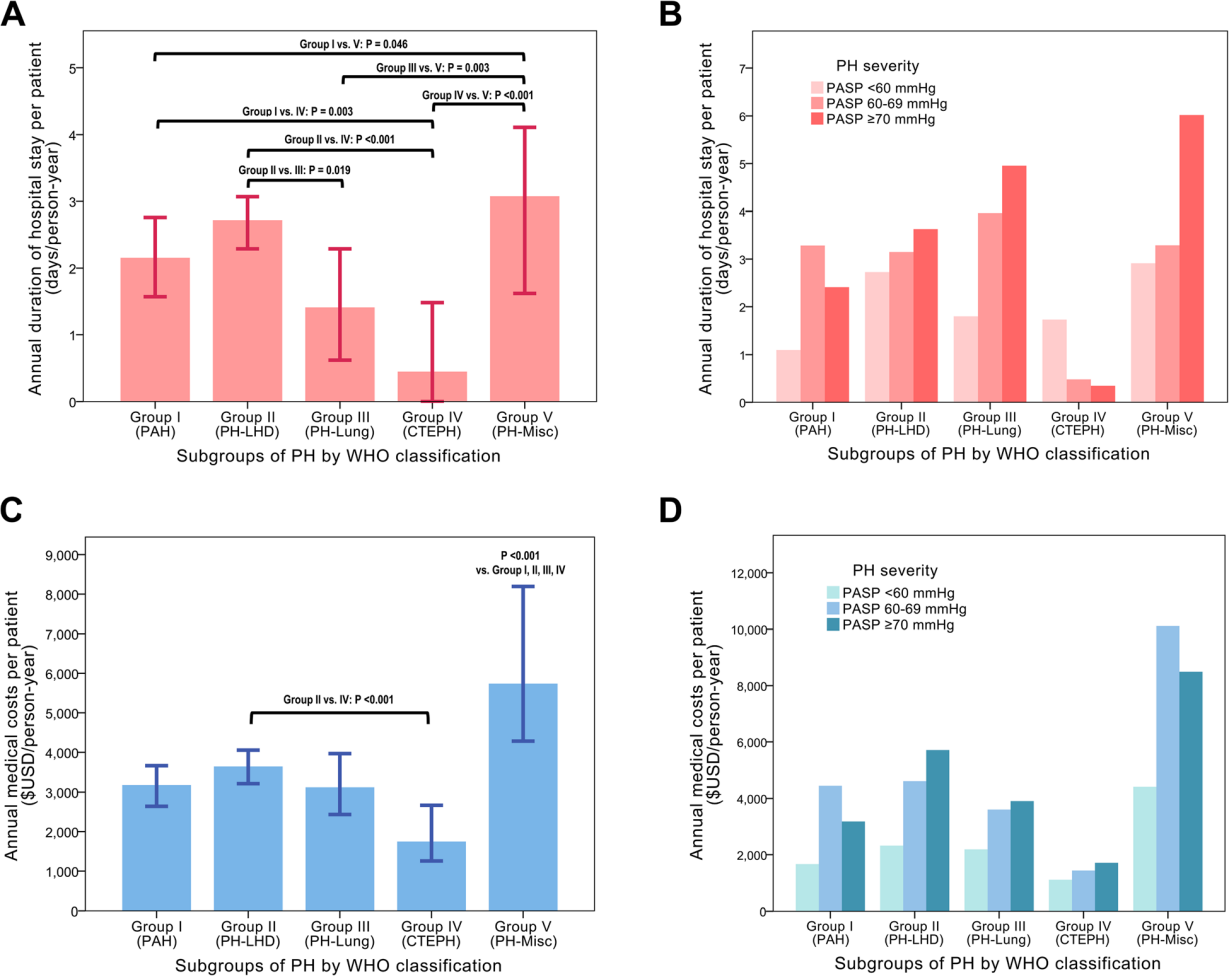
Healthcare utilization and medical expenditure across the subgroups of PH. Durations of hospital stay per person-year according to the (**a**) PH subgroups, and (**b**) severity of PH among the subgroups. Total medical expenditure per person-year was also compared according to the (**c**) PH subgroups, and (**d**) severity of PH among the subgroups. Patients with follow-up duration < 6 months were excluded from this analysis

During the follow-up period, there were 6692 events of hospitalizations with 54,602 days of hospital stay and 52,735 OPD visits among the total study population. The annual number of hospitalizations and duration of hospital stay were significantly higher in group V and group II than other groups (Fig. 3a and Table 3). There was a tendency for a longer hospitalization among those with higher PASP in groups I, II, and V (Fig. 3b).

**Table 3:** Healthcare utilization and medical expenditures in PH subgroups

	Total study population (*n* = 2185)	Group I (PAH) (*n* = 333)	Group II (PH-LHD) (*n* = 1189)	Group III (PH-Lung) (*n* = 335)	Group IV (CTEPH) (*n* = 102)	Group V (PH-Miscellaneous) (*n* = 226)
Healthcare utilization						
Number of hospitalizations	1 (0–4)	2 (0–4)	1 (0–4)	1 (0–3)	1 (0–2)	2 (1–6)
Total duration of hospitalization (days)	9 (0–29)	9 (0–27)	10 (0–30)	4 (0–24)	2 (0–13)	9 (1–42)
Number of OPD visits	15 (4–34)	22 (9–43)	15 (6–33)	7 (1–23)	12 (2–27)	19 (5–43)
Annualized rates of healthcare utilization						
Number of hospitalizations per year (events/person-year)	0.4 (0.0–1.2)	0.4 (0.0–1.1)	0.4 (0.0–1.2)	0.3 (0.0–1.1)	0.2 (0.0–0.6)	0.7 (0.2–1.6)
Duration of hospitalization per year (days/person-year)	2.3 (0.0–8.2)	2.2 (0.0–6.8)	2.7 (0.0–8.3)	1.4 (0.0–8.9)	0.5 (0.0–4.9)	3.1 (0.4–10.9)
Number of OPD visits per year (visits/person-year)	4.6 (1.4–9.4)	5.5 (2.6–10.0)	4.6 (1.7–9.2)	2.5 (0.4–8.2)	4.0 (0.8–8.4)	6.0 (1.8–12.1)
Medical expenditure^a^						
Total costs ($USD)	12,184 (3028–34,132)	13,564 (4247–30,750)	12,763 (2586–34,549)	8086 (1834–27,482)	5449 (2436–12,618)	20,971 (5767–68,537)
Cost for testing ($USD)	4050 (1668–8560)	4256 (2036–8311)	4282 (1609–8706)	3290 (1427–7127)	2684 (1597–5630)	5057 (1992–12,610)
Cost for medications ($USD)	422 (129–1272)	598 (176–1935)	409 (123–1187)	296 (119–823)	283 (93–950)	493 (155–1868)
Annual medical expenditure						
Total costs per year ($USD/person-year)	3531 (949–10,708)	3179 (1175–7910)	3641 (829–10,924)	3123 (792–10,092)	1749 (808–5181)	5739 (2094–20,048)
Cost for testing per year ($USD/person-year)	1135 (452–2492)	1017 (488–2072)	1196 (440–2490)	1070 (393–2397)	785 (366–1735)	1573 (594–3183)
Cost for medications per year ($USD/person-year)	110 (34–372)	140 (42–479)	105 (31–348)	95 (35–298)	81 (25–238)	139 (43–690)

Data are shown as median (interquartile ranges; Q1-Q3)

^a^Medical costs were assessed in Korean Won (KRW), and were translated into US dollars ($USD) using a currency exchange rate of 1123 KRW/$USD

Healthcare utilization was compared between the PH subgroups using the number of hospitalization, total duration of hospital stay, and number of outpatient department (OPD) visits. Medical expenditure was compared using the total medical costs, cost for testing, and cost for medications. The outcome parameters were annualized and averaged within each PH subgroup

Abbreviations: same as Table 1

The annual medical expenditure was 3531 $USD/person-year (IQR, 949–10,708) in total study population, and showed significant difference across the subgroups of PH: the total cost per year was significantly larger in group V, followed by group II (Fig. 3c and Table 3). Patients with higher PASP had more medical expenditure than those with lower PASP in most subgroups of PH (Fig. 3d).

## Discussion

We identified patients with PH among those referred for echocardiography, and categorized the patients according to the WHO classification. The five diagnostic subgroups of PH demonstrated different clinical characteristics and prognosis. Risk factors for mortality were different between the subgroups; however, the severity of PH was associated with higher risk of mortality in most subgroups. Medical expenditure and healthcare utilization were also different between the subgroups, but tended to be proportional to the PH severity.

### Different clinical features and prognosis between PH subgroups

Group I (PAH) patients were younger females, and had less traditional coronary risk factors compared to other PH groups, as reported in previous studies [6]. In this subgroup, major risk factors for mortality were age, lower hemoglobin level, renal dysfunction and the severity of PH, whereas traditional coronary risk factors and LV function parameters were not associated with mortality risk. It has been suggested that the major determinant of clinical deterioration in group I PH is the increase in pulmonary artery pressure through vascular remodeling, and this process is related with anemia and renal dysfunction [9–11]. Better outcomes were observed in PAH with congenital shunt, and worse outcomes in those with connective tissue disease or idiopathic PAH. These findings are similar to the previous studies, [4, 12] and suggest the importance of underlying pathophysiology.

Group II PH (PH-LHD) was characterized with impaired LV systolic and/or diastolic function [13]. Typical features of HF, such as depressed LVEF and dilated LV cavity, showed associations with mortality in this subgroup. Presence of PH was an independent prognostic factor for mortality, which was also observed in previous studies [14]. The association between the low BMI and low mortality risk support the obesity paradox in HF [15].

In group III PH (PH-Lung), the risk factors for all-cause mortality were age, lower level of hemoglobin, and the severity of PH, whereas the presence of HTN was associated with a lower mortality risk. Indeed, anemia is prevalent in patients with chronic obstructive pulmonary disease (COPD) by multi-factorial pathogenesis, including chronic inflammation, nutritional deficits, stress ulcer, and effects of cigarette smoking [16, 17]. The presence of anemia is associated with increased mortality risk, and notably, may contribute to the development and progression of PH in COPD patients through hypoxic pulmonary vasoconstriction [18]. Our findings support that lower hemoglobin level and elevated PASP may reflect a more advanced stage of PH-Lung, which is associated with an increased risk of all-cause mortality [19, 20]. Of note, the risk of all-cause mortality was the highest in the group III, and lowest in the group II, among the PH subgroups, in concordance with the results from the ASPIRE (Assessing the Spectrum of Pulmonary hypertension Identified at a REferral centre) registry, where the 5-year survival rates were less than 40% among the group III PH patients, about 50% among the group I PH patients, and higher than 60% among the group II PH patients [4]. However, according to the Armadale cohort, the risk of mortality was similar between the patients with PH-LHD (group II) and PH-Lung (group III), whereas lower in PAH (group I) [2]. These differences may be attributable to the severity of PH of each study: the Armadale cohort included the patients with mild PH (inclusion criteria, PASP > 40 mmHg; mean PASP, 56 mmHg), whereas we mainly enrolled those with moderate or severe PH (inclusion criteria, peak TR velocity > 3.4 m/sec; mean PASP, 66 mmHg).

The significant difference in prognosis between PH-LHD and PH-Lung, despite the similar severity of PH, reflects the different etiology for development and progression of PH in each subgroup. In patients with HF, diastolic dysfunction leads to passive backward transmission of LV filling pressures, and some of these patients develop further increment in pulmonary artery pressure through pulmonary vasoconstriction, decreased nitric oxide availability, increased endothelin expression, and vascular remodeling [21, 22]. Given the elevated LV end-diastolic pressure (LVEDP) as the main pathophysiology in PH-LHD, there is a room for alleviation of the increased LV filling pressure and PH through intravascular volume reduction, as suggested in previous studies with findings of the exaggerated relationship between volume overload and the increase in LVEDP and PAP in HF patients [9, 10]. In contrast, PH in chronic lung disease is attributable to the fibrotic injuries including neomuscularization, intimal thickening, and medial hypertrophy through chronic hypoxia, cigarette smoking, and airway and vascular wall inflammation [21]. Because of these irreversible structural changes, the presence of PH indicates far-advanced stage of pulmonary vascular remodeling. Indeed, the prevalence of PH is approximately 90% in stage IV COPD, and 5-year survival rate of patients with COPD and PH is only 36% [19, 23]. Therefore, the differences in pathophysiology between the group II and group III PH seem to be the main reason for the different prognosis.

In our study, most of the patients in group V (PH-Miscellaneous) had CKD/ESRD. It would be attributable to the study design: the study population was identified from tertiary referral centers, and the patients with prior or current malignancies were excluded. According to the previous studies, the prevalence of PH has been reported to range from 10 to 30% in CKD/ESRD patients [24]. Development of PH in these patients is explained by increased cardiac output, anemia, uremic physiology, vascular calcification, and recurrent subclinical thromboembolic events [24]. Of note, there is a debate regarding the elevated LVEDP as a potential cause of PH in CKD/ESRD. Considering that patients with CKD/ESRD and PH usually have elevated LV filling pressure, it can be argued that some of these patients could have been classified as group II (PH-LHD) [24, 25]. Although the patients with ≥grade II diastolic dysfunction were categorized into PH-LHD regardless of the presence of CKD/ESRD in the present study, the overall echocardiographic parameters of group V were similar to those in group II. Our findings suggest that diastolic dysfunction maybe an important factor for development of PH in CKD/ESRD, and that the differential categorization between group II and group V can be difficult as those groups share similar pathophysiology. Although the group II PH patients and the group V PH patients with CKD/ESRD have similar characteristics of volume overload and LV diastolic dysfunction, the healthcare utilization and medical expenditure were larger in group V patients than in group II patients. These findings may be attributable to the additional medications for renal dysfunction and more hospital visits for dialysis in the group V patients with CKD/ESRD.

### PH as a marker of “patient-burden”

The annual medical expenditure was the largest in group V, followed by group II. Frequency of healthcare utilization showed similar results. Our findings suggest that different patterns of healthcare utilization and medical expenditure may originate from the differences in pathophysiology and clinical consequences. Although patients with PAH are recognized as consuming the highest medical expenditure, we showed that medical expenditure in group V PH can be larger than those of group I PH. It might be attributable to the following factors: (1) about 70% of the group I patients had ever received PAH-specific medications as a clinical trial and could not be identified from reimbursement data; (2) the prescription of PAH-specific drugs was under strict criteria for insurance coverage; and (3) most of group V patients had ESRD, which requires large medical expenditures.

In usual clinical situation, the presence of PH and its severity can be recognized as a secondary finding to a specific pathophysiology attributable to the development and progression of PH. However, we demonstrated that the severity of PH per se may affect the mortality risk as well as the healthcare utilization and medical expenditure regardless of the WHO classification. Therefore, the presence and severity of PH should also be recognized with sufficient clinical vigilance.

### Limitations

First, the presence of PH was determined by echocardiography rather than cardiac catheterization. Therefore, we could not provide invasive measurements of the pulmonary artery pressure and the differentiation between pre-capillary and post-capillary PH. Given the lack of cardiac catheterization data, we tried to improve the specificity for the diagnosis of PH using a high cutoff value of TR Vmax. According to the clinical guidelines, patients with a TR Vmax > 3.4 m/sec are highly suggestive of PH and do not require any further echocardiographic signs for the diagnosis of PH [3]. This inclusion criterion would have contributed to the exclusion of patients with transient elevation of PASP from other causes rather than true PH. Although the diagnosis of PH of our study can be inaccurate in some patients, the overall results would not be changed because the cutoff value of TR velocity (3.4 m/sec) was high enough to select the patients with PH [3]. Second, we could not provide the symptomatic status due to the incomplete medical records. Given the well-established prognostic value of symptomatic status in PH patients, the interpretation of our findings needs caution. Third, we compared the clinical characteristics and the profiles of risk factors for mortality between the PH subgroups, except for group IV PH in which the sample size was not large enough. Fourth, most of the group V PH patients had CKD/ESRD, and there were few patients with other diseases. However, we think the distribution of WHO groups of our study can reflect the real-world practice.

## Conclusions

The five diagnostic subgroups of PH by the WHO classification had significantly different clinical characteristics, echocardiographic findings, and the risk of mortality. The profiles of risk factors for mortality were different according to the etiology of PH, while the elevated PASP was associated with higher mortality risk in most subgroups. Medical expenditure and healthcare utilization were also different between the PH subgroups, but tended to be proportional to the severity of PH. Our findings implicate the importance of comprehensive understanding of the PH in each subgroup.

## Supplementary information

**Additional file 1: **Diagnostic codes for classification of PH subgroups**.** A table of diagnostic codes for the classification of PH subgroups.

**Additional file 2:** Univariable Cox proportional hazard regression analysis. A table of results of univariable Cox proportional hazard regression analyses for the occurrence of all-cause mortality in total study population.

**Additional file 3:** Relationship between the hemoglobin levels and the risk of all-cause mortality. A spline curve showing the relationship between the levels of hemoglobin and the risk of all-cause mortality in total study population.

## Data Availability

The datasets used and/or analyzed during the current study are available from the corresponding author on reasonable request.

## Abbreviations

CKD: Chronic kidney disease
CTEPH: Chronic thromboembolic pulmonary hypertension
ESRD: End-stage renal disease
LVEF: Left ventricular ejection fraction
PAH: Pulmonary arterial hypertension
PAP: Pulmonary artery pressure
PASP: Pulmonary artery systolic pressure
PH: Pulmonary hypertension
PH-LHD: Pulmonary hypertension due to left-sided heart disease
PH-Lung: Pulmonary hypertension due to lung disease or hypoxia
PH-Miscellaneous: Pulmonary hypertension with multifactorial mechanisms
TR: Tricuspid regurgitation
WHO: World Health Organization
