# Supplementary material for: Healthcare utilization, medical expenditure, and mortality in Korean patients with pulmonary hypertension
Source: BMC Pulm Med. 2019 Oct 30;19:189. doi: 10.1186/s12890-019-0945-0 (PMC6822398; doi:10.1186/s12890-019-0945-0)
Supplement: Supplementary file 1 — Additional file 1: Diagnostic codes for classification of PH subgroups. A table of diagnostic codes for the classification of PH subgroups. [file 12890_2019_945_MOESM1_ESM.docx]

**Additional file 1. Diagnostic codes for classification of PH subgroups**

| **PH groups** | **Disease entity** | **ICD diagnosis** | **ICD diagnostic code** |
| --- | --- | --- | --- |
| Group I PH  (PAH) | Idiopathic PAH | Primary pulmonary hypertension | I27.0 |
|  | Familial PAH | Primary pulmonary hypertension | I27.0 |
|  | Associated with: | Other secondary pulmonary hypertension | I27.2 |
|  | Connective tissue disease | Seropositive rheumatoid arthritis  Other rheumatoid arthritis  Lung disease in rheumatoid arthritis | M05  M06  J99.0 |
|  |  | Polyarteritis nodosa and related conditions | M30 |
|  |  | Other necrotizing vasculopathies | M31 |
|  |  | Lupus erythematosus Systemic lupus erythematosus | L93  M32 |
|  |  | Dermatomyositis  Polymyositis  Dermatopolymyositis, unspecified | M33.0, M33.1  M33.2  M33.9 |
|  |  | Systemic sclerosis  Localized scleroderma  Linear scleroderma | M34  L94.0  L94.1 |
|  |  | Other systemic involvement of connective tissue disease  - Sicca syndrome (Sjogren)  - Other overlap syndrome  - Behcet disease | M35  M35.0  M35.1  M35.2 |
|  | HIV infection | HIV disease resulting in infectious and parasitic diseases  HIV disease resulting in other specified diseases  HIV disease resulting in other conditions  Unspecified HIV disease  Asymptomatic HIV infection status  Laboratory evidence of HIV | B20  B22  B23  B24  Z21  R75 |
|  | Portal hypertension | Portal hypertension  Alcoholic liver disease  Toxic liver disease  Hepatic failure, not elsewhere classified  Chronic hepatitis, not elsewhere classified  Fibrosis and cirrhosis of liver  Chronic viral hepatitis  Unspecified viral hepatitis | K76.6  K70  K71  K72  K73  K74  B18  B19 |
|  | Congenital heart disease | Congenital malformations of cardiac chambers and connections  Congenital malformations of cardiac septa  - Eisenmenger defect  Congenital malformations of aortic and mitral valves  Other congenital malformations of heart  Congenital malformations of great arteries  Congenital malformations of great veins | Q20  Q21  Q21.8  Q23  Q24  Q25  Q26 |
|  | Schistosomiasis | Schistosomiasis  Pulmonary heart disease in schistosomiasis | B65  I52.1 |
| Group II PH  (PH-LHD) | LV dysfunction (systolic, diastolic) | Heart failure  Cardiomyopathy  Pulmonary edema | I50  I42  J81 |
|  | Valvular disease | Rheumatic mitral valve diseases  Rheumatic aortic valve diseases  Rheumatic tricuspid valve disease  Multiple valve diseases  Nonrheumatic mitral valve disorders  Nonrheumatic aortic valve disorders  Mitral valve disorders in diseases classified elsewhere  Aortic valve disorders in diseases classified elsewhere  Multiple valve disorders in diseases classified elsewhere | I05  I06  I07  I08  I34  I35  I39.0  I39.1  I39.4 |
| Group III PH  (PH-Lung) | COPD  Other pulmonary diseases with mixed restrictive and obstructive pattern | Bronchitis, not specified as acute or chronic  Simple and mucopurulent chronic bronchitis  Unspecified chronic bronchitis  Emphysema  Other chronic obstructive pulmonary disease  Asthma  Status asthmaticus  Bronchiectasis  Coal-worker pneumoconiosis  Pneumoconiosis due to asbestos and other mineral fibers  Pneumoconiosis due to dust containing silica  Pneumoconiosis due to other inorganic dusts  Unspecified pneumoconiosis  Pneumoconiosis associated with tuberculosis  Airway disease due to specific organic dust  Hypersensitivity pneumonitis due to organic dust  Respiratory conditions due to inhalation of chemicals, gases, fumes and vapors  Pneumonitis due to solids and liquids  Respiratory conditions due to other external agents  Kyphoscoliotic heart disease | J40  J41  J42  J43  J44  J45  J46  J47  J60  J61  J62  J63  J64  J65  J66  J67  J68  J69  J70  I27.1 |
|  | Interstitial lung disease | Other interstitial pulmonary diseases | J84 |
|  | Sleep-disordered breathing | Sleep apnea | G47.3 |
|  | Alveolar hypoventilation disorders | Extreme obesity with alveolar hypoventilation | E66.2 |
|  | Chronic exposure to high altitude | Other and unspecified effects of high altitude  Exposure to high and low air pressure and changes in air pressure | T70.2  W94 |
| Group IV PH  (CTEPH) | Pulmonary embolism | Pulmonary embolism | I26 |
|  | Parasites (hydratidosis) | Echinococcosis, other and unspecified | B67.9 |
| Group V PH  (PH-Miscellaneous) | Hematologic disorders | Anemia due to enzyme disorders  Thalassemia  Sickle-cell disorders  Other hereditary hemolytic anemias  Acquired hemolytic anemia | D55  D56  D57  D58  D59 |
|  |  | Chronic myeloproliferative disease | D47.1 |
|  |  | Splenectomy (status post splenectomy) | D73.0 |
|  | Systemic disorders | Sarcoidosis | D86 |
|  |  | Lymphangioleiomyomatosis | D18.1 |
|  |  | Neurofibromatosis | Q85.0 |
|  | Metabolic disorders | Glycogen storage diseases | E74.0 |
|  |  | Other sphingolipidosis: Fabry, Gaucher, Niemann-Pick, etc | E75.2 |
|  |  | Congenital iodine-deficiency syndrome  Iodine-deficiency-related thyroid disorders and allied conditions  Subclinical iodine-deficiency hypothyroidism  Other hypothyroidism  Other nontoxic goiter  Thyrotoxicosis (hyperthyroidism)  Thyroiditis  Other disorders of thyroid | E00  E01  E02  E03  E04  E05  E06  E07 |
|  | Others | Thrombotic microangiopathy  Chronic kidney disease | M31.1  N18, N19 |

International Statistical Classification of Diseases and Related Health Problems 10th Revision (ICD-10) Version was used.

Diagnostic codes of malignant diseases were not included, because the patients with prior or current malignancy were excluded from the study population.

Abbreviations: PH, pulmonary hypertension; PAH, pulmonary arterial hypertension; HIV, human immunodeficiency virus; LHD, left heart disease; COPD, chronic obstructive pulmonary disease; ICD, International Statistical Classification of Diseases and Related Health Problems;
