# Supplementary material for: Healthcare utilization, medical expenditure, and mortality in Korean patients with pulmonary hypertension
Source: BMC Pulm Med. 2019 Oct 30;19:189. doi: 10.1186/s12890-019-0945-0 (PMC6822398; doi:10.1186/s12890-019-0945-0)
Supplement: Supplementary file 2 — Additional file 2: Univariable Cox proportional hazard regression analysis. A table of results of univariable Cox proportional hazard regression analyses for the occurrence of all-cause mortality in total study population. [file 12890_2019_945_MOESM2_ESM.docx]

**Additional file 2. Univariable Cox proportional hazard regression analysis**

|  | **Unadjusted HR** | **95% CI** | **P value** |
| --- | --- | --- | --- |
| **Age (per 10 years)** | 1.28 | 1.213 – 1.352 | <0.001 |
| **Age ≥60 years** | 2.108 | 1.735 – 2.562 | <0.001 |
| **Age ≥70 years** | 1.907 | 1.641 – 2.217 | <0.001 |
| **Male sex** | 1.565 | 1.353 – 1.810 | <0.001 |
| **BMI (kg/m^2^)** | 0.955 | 0.928 – 0.983 | 0.002 |
| **BMI >22.5 kg/m^2^ (median)** | 0.725 | 0.586 - 0.897 | 0.003 |
| **BMI >25 kg/m^2^** | 0.723 | 0.556 – 0.940 | 0.015 |
| **BMI >30 kg/m^2^** | 0.782 | 0.440 – 1.392 | 0.404 |
| **BSA (m2)** | 0.59 | 0.539 – 0.646 | <0.001 |
| **Comorbidities** |  |  |  |
| **HTN** | 0.896 | 0.764 – 1.051 | 0.176 |
| **DM** | 1.074 | 0.918 – 1.256 | 0.375 |
| **Dyslipidemia** | 0.9 | 0.725 – 1.117 | 0.34 |
| **CKD** | 1.236 | 1.055 – 1.449 | 0.009 |
| **Chronic hepatitis** | 0.678 | 0.168 – 2.734 | 0.585 |
| **Laboratory findings** |  |  |  |
| **Hemoglobin (g/dL)** | 0.908 | 0.882 – 0.936 | <0.001 |
| **Anemia*** | 1.865 | 1.588 – 2.189 | <0.001 |
| **Total cholesterol (mg/dL)** | 0.992 | 0.990 – 0.994 | <0.001 |
| **Triglyceride (mg/dL)** | 0.998 | 0.996 – 1.000 | 0.031 |
| **HDL-cholesterol (mg/dL)** | 0.997 | 0.989 – 1.004 | 0.354 |
| **LDL-cholesterol (mg/dL)** | 0.992 | 0.989 – 0.996 | <0.001 |
| **BUN (mg/dL)** | 1.006 | 1.003 – 1.010 | <0.001 |
| **Creatinine (mg/dL)** | 1.001 | 0.975 – 1.029 | 0.913 |
| **GFR by MDRD (mL/min/1.73m^2^)** † | 0.997 | 0.996 – 0.999 | <0.001 |
| **GFR <60 mL/min/1.73m^2^** | 1.502 | 1.297 – 1.739 | <0.001 |
| **HbA1c (%)** | 1.077 | 0.995 – 1.164 | 0.065 |
| **Echocardiographic findings** |  |  |  |
| **LVEDD (mm)** | 0.987 | 0.980 – 0.995 | 0.001 |
| **LVEDD >55 mm** | 0.872 | 0.748 - 1.018 | 0.082 |
| **LVESD (mm)** | 0.992 | 0.985 – 0.999 | 0.019 |
| **LVEDV (mL)** | 1.004 | 1.002 – 1.005 | <0.001 |
| **LVEDV >100 mL** | 1.643 | 1.293 - 2.088 | <0.001 |
| **LVESV (mL)** | 1.005 | 1.003 – 1.006 | <0.001 |
| **LVEF (%)** | 0.998 | 0.994 – 1.003 | 0.489 |
| **LVMI (g/m^2^)** | 0.997 | 0.994 – 0.999 | 0.013 |
| **LA dimension >40 mm** | 0.824 | 0.704 – 0.965 | 0.016 |
| **LAVI >34 mL/m^2^** | 0.934 | 0.629 - 1.388 | 0.736 |
| **LAVI >60 mL/m^2^** | 0.96 | 0.721 - 1.279 | 0.782 |
| **Mitral inflow** |  |  |  |
| **E velocity (m/sec)** | 0.758 | 0.643 - 0.893 | 0.001 |
| **A velocity (m/sec)** | 1.009 | 1.000 - 1.018 | 0.057 |
| **E/A ratio** | 0.827 | 0.745 - 0.917 | <0.001 |
| **Deceleration time (msec)** | 0.999 | 0.998 - 1.000 | 0.025 |
| **Mitral annular TDI** |  |  |  |
| **e' velocity (cm/sec)** | 0.94 | 0.905 - 0.976 | 0.001 |
| **a' velocity (cm/sec)** | 1.041 | 1.015 - 1.068 | 0.002 |
| **s' velocity (cm/sec)** | 0.995 | 0.966 - 1.025 | 0.737 |
| **E/e' ratio** | 0.995 | 0.988 – 1.001 | 0.109 |
| **E/e' ratio >15** | 0.893 | 0.761 – 1.048 | 0.165 |
| **PASP (mmHg)** | 1.011 | 1.006 - 1.016 | <0.001 |
| **PASP >62 mmHg (median)** | 1.451 | 1.254 - 1.679 | <0.001 |
| **PASP <60 mmHg (reference)** | *Referenced* |  |  |
| **PASP 60-70 mmHg** | 1.6 | 1.346 - 1.901 | <0.001 |
| **PASP ≥70 mmHg** | 1.837 | 1.527 - 2.211 | <0.001 |

Unadjusted hazard ratios (HR) for all-cause mortality were obtained using the Cox proportional hazard regression model analysis. Continuous variables, such as age, BMI, GFR, PASP and other echocardiographic parameters were categorized using the median or clinical relevant values.

* Anemia was defined as hemoglobin levels <13 g/dL for men and <12 g/dL for women.

† GFR was calculated using the Modification of Diet in Renal Disease (MDRD) equation.

Abbreviations: BMI, body-mass index; BSA, body surface area; HTN, hypertension; DM, diabetes mellitus; CKD, chronic kidney disease; HDL, high-density lipoprotein; LDL, low-density lipoprotein; BUN, blood urea nitrogen, GFR, glomerular filtration rate; HbA1c, hemoglobin A1c; LVEDD, left ventricular end-diastolic dimension; LVESD, left ventricular end-systolic dimension; LVEDV, left ventricular end-diastolic volume; LVESV, left ventricular end-systolic volume; LVEF, left ventricular ejection fraction; LVMI, left ventricular mass index; LA, left atrium; LAVI, left atrial volume index; TDI, tissue Doppler imaging; TR, tricuspid regurgitation; PASP, pulmonary artery systolic pressure.
