# Supplementary material for: Healthcare utilization, medical expenditure, and mortality in Korean patients with pulmonary hypertension
Source: BMC Pulm Med. 2019 Oct 30;19:189. doi: 10.1186/s12890-019-0945-0 (PMC6822398; doi:10.1186/s12890-019-0945-0)
Supplement: Supplementary file 3 — Additional file 3: Relationship between the hemoglobin levels and the risk of all-cause mortality. A spline curve showing the relationship between the levels of hemoglobin and the risk of all-cause mortality in total study population. [file 12890_2019_945_MOESM3_ESM.docx]

**Additional file 3. Relationship between the hemoglobin levels and the risk of all-cause mortality**


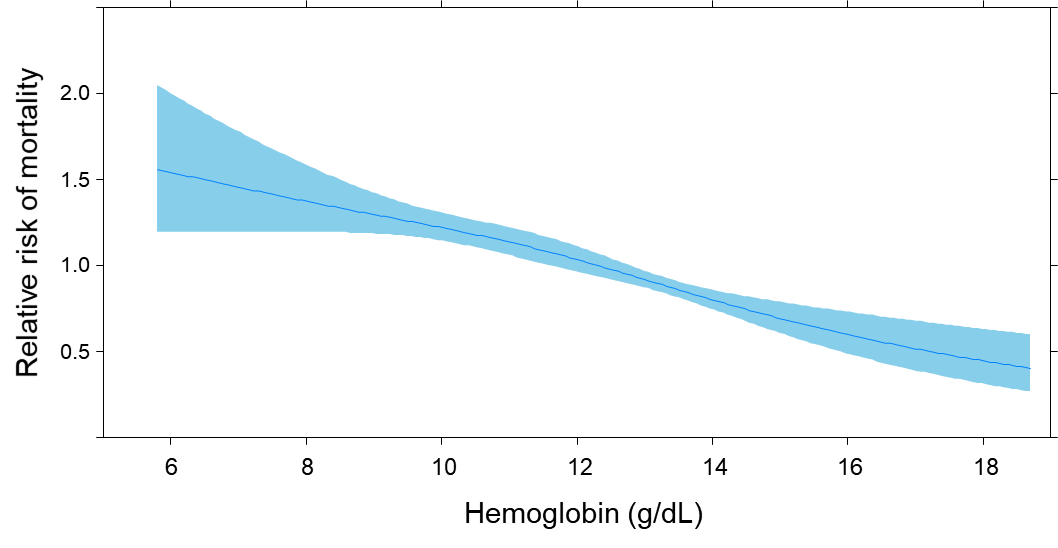


The levels of hemoglobin showed an inverse linear relationship with the risk of all-cause mortality in total study population of patients with pulmonary hypertension.
